# Outcomes of Stereotactic Body Radiotherapy for Metastatic Colorectal Cancer With Oligometastases, Oligoprogression, or Local Control of Dominant Tumors

**DOI:** 10.3389/fonc.2020.595781

**Published:** 2021-01-29

**Authors:** Xiaoqin Ji, Yulu Zhao, Xixu Zhu, Zetian Shen, Aomei Li, Cheng Chen, Xiaoyuan Chu

**Affiliations:** ^1^ Department of Radiation Oncology, Jinling Hospital, Nanjing Clinical School of Nanjing Medical University, Nanjing, China; ^2^ Department of Medical Oncology, Jinling Hospital, Nanjing Clinical School of Nanjing Medical University, Nanjing, China

**Keywords:** colorectal cancer, stereotactic body radiotherapy, oligometastases, oligoprogression, cyberknife

## Abstract

**Aim:**

To evaluate the clinical outcomes of metastatic colorectal cancer (mCRC) patients with oligometastases, oligoprogression, or local control of dominant tumors after stereotactic body radiotherapy (SBRT) and establish a nomogram model to predict the prognosis for these patients.

**Methods and Materials:**

A cohort of 94 patients with 162 mCRC metastases was treated with SBRT at a single institution. Treatment indications were oligometastases, oligoprogression, and local control of dominant tumors. End points of this study were the outcome in terms of progression-free survival (PFS), overall survival (OS), local progression (LP), and cumulative incidence of starting or changing systemic therapy (SCST). In addition, univariate and multivariable analyses to assess variable associations were performed. The predictive accuracy and discriminative ability of the nomogram were determined by concordance index (C-index) and calibration curve.

**Results:**

Median PFS were 12.6 months, 6.8 months, and 3.7 months for oligometastases, oligoprogression, and local control of dominant tumors, respectively. 0-1 performance status, < 10 ug/L pre-SBRT CEA, and ≤ 2 metastases were significant predictors of higher PFS on multivariate analysis. Median OS were 40.0 months, 26.1 months, and 6.5 months for oligometastases, oligoprogression, and local control of dominant tumors, respectively. In the multivariate analysis of the cohort, the independent factors for survival were indication, performance status, pre-SBRT CEA, and PTV, all of which were selected into the nomogram. The calibration curve for probability of survival showed the good agreement between prediction by nomogram and actual observation. The C-index of the nomogram for predicting survival was 0.848.

**Conclusions:**

SBRT for metastases derived from colorectal cancer offered favorable survival and symptom palliation without significant complications. The proposed nomogram could provide individual prediction of OS for patients with mCRC after SBRT.

## Introduction

Colorectal cancer (CRC) is a major cause of cancer-related deaths, with a 5-year survival rate of 64% (1). 21% of patients are diagnosed with metastasis, and approximately 50% of patients with colorectal cancer in due course of time will develop distant metastasis, and the 5-year survival rate is less than 14% (1, 2). The most common site of CRC metastasis is the liver, followed by the lungs and bones (3–5). Systemic therapy is the main treatment for patients with metastatic colorectal cancer (mCRC). With the introduction of new chemotherapy regimens, targeted therapies and immunotherapy, the efficacy of systemic therapy has been improved. Local treatment can be used to reduce the burden of tumors to better control the disease, thereby improving the overall survival (OS).

Hellmann and Weichselbaum proposed an intermediate clinical state between widespread metastases and locoregionally confined malignancy in 1995, called oligometastases (6, 7). Oligometastatic disease is manifested by the presence of limited metastases in limited organs. For patients with oligometastases of colorectal cancer, intensive treatment of metastases has improved OS (8, 9). In surgically resected liver metastasis, the 5-year OS rate of CRC patients was between 50 and 60% (10–12). However, in some cases, many patients with metastases cannot be treated by surgery due to larger tumor size and bad location. Thus, other local treatment methods should be considered. Over the past two decades, extensive clinical experience has proved that SBRT is a non-invasive, high-precision technical method. It could deliver ablative treatments for different metastatic sites (liver, lung, brain, bone/spine, adrenal, lymphadenopathy, pancreas, etc.) (13–15) with little impact on acute quality of life. Compared to surgery, SBRT has advantages of lower morbidity, good immediate tolerance and no need for general anesthesia. SBRT could not only serve as an alternative to surgery, but also a complementary treatment. It can shrink the lesion to achieve resectability of the tumor. In addition, SBRT can eliminate residual lesions or positive margins after surgical resection, reducing the risk of local recurrence. Many non-random studies of oligometastases with SBRT have achieved a local control rate of 80%, and the progression-free survival (PFS) of two to five years was about 20% (16).

In addition to oligometastases, the use of SBRT for oligoprogression has attracted increasing attention. In this case, one or several metastatic lesions are growing, and other lesions are stable under systemic treatment strategies (17). Progressive tumors treated with SBRT may delay the start or change need for systemic therapy. This may have clinical benefits, including improved PFS, OS and life quality of patients (18–20). Local control of dominant tumors is another increasing indication for SBRT (21), a clinical situation where the local tumor may cause severe morbidity, obvious pain or obstruction symptoms. In this case, the main goal of SBRT targeting dominant tumors is to alleviate symptoms.

Most studies included SBRT in patients with oligometastatic or oligoprogressive cancers irrespective of histology (18, 22, 23), which could not determine the specific benefits of SBRT for specific cancer histology (24). In this study, we analyzed the clinical outcomes of mCRC patients with oligometastases, oligoprogression, or local control of dominant tumors after SBRT and established a nomogram model to predict the prognosis for these patients.

## Methods and Materials

### Patients

Ninety-four patients with histologically confirmed colorectal adenocarcinoma underwent radical surgery regardless of whether they received adjuvant chemotherapy, and were diagnosed with metastatic or synchronous metastasis. They underwent SBRT from January 2010 to December 2018 in Jinling Hospital affiliated to Nanjing Medical University (Nanjing, China). The retrospective study was approved by our institutional Research Ethics Board. The criteria for patients undergoing SBRT were as follows: (1) oligometastases, with the maximum of 5 metastases (≤5 cm in size) diagnosed in the maximum of 2 sites. (2) oligoprogression, only irradiating the growing tumor (≤ 5 growing tumors), and all other lesions are stable. (3) local control of dominant tumors, and clinically hope to alleviate symptoms or prevent anticipated complications of progression, even if other tumors were progressing. The last indication refers to the situation where the local tumor may cause severe morbidity, obvious pain or obstruction symptoms. The main exclusion criteria included prior history of malignant tumors in other areas and prior in-field radiotherapy.

### Techniques of SBRT

In our study, SBRT was delivered using CyberKnife (Accuray Incorporated, Sunnyvale, CA, USA). For metastatic lesions located in internal moving organs (such as lung metastases, liver metastases, and abdominal metastatic lymph nodes), gold fiducial tumor markers were implanted under ultrasound or CT guidance before SBRT. The gold fiducials need to be placed in or near the lesion. For patients with poor physical conditions or the location of tumors near large blood vessels, which are more at risk of repeated punctures, 1–2 gold fiducials were embedded. Other tolerable patients received 3–4 implants. The gold fiducial is a 99% pure gold cylinder with a length of 5 mm and a diameter of 0.8 mm. To ensure that the position of the gold labels relative to the tissues are stable, the CT positioning scan was generally conducted one week after the gold fiducials were embedded. Different methods were used to track the lesions at different sites. Intracranial tumors were tracked using six-dimensional skull tracking, and spinal metastases were tracked using XSight spine tracking approach. For thoracic and abdominal soft tissue tumors, respiration synchronous tracking (Synchrony) was used to track the movement of the fiducials instead of tumor movements for simultaneous irradiation.

During the Body CT (Brilliace Big Bore 16CT Philips Germany) simulation positioning, the patient was fixed with a vacuum pad. Simultaneously, intravenous contrast was injected to clearly show the tumors. The patient breathed normally before the CT scan and held the breath at the end of the inhalation to scan. The CT scan range is 15 cm above and below the lesion, with a layer thickness of 1 mm. Patients with brain metastases were fixed with a thermoplastic mask. The gross tumor volume (GTV) was delineated on simulation CT imaging, and co-registered with MRI scan or PET-CT scan (if available). According to disease site and dimensions, a margin of 0-5 mm was added to GTV to form the planning target volume (PTV). When evaluating the CyberKnife treatment plans, the normal tissue constraints and dose prescription points were as per Radiation Therapy Oncology Group (RTOG)/NRG SBRT protocols, and published dose-volume constraint tables for hypofractionation (25, 26).

SBRT was usually executed one time a day and five days a week. SBRT usually takes about one hour. Therefore, it is difficult for patients with severe pain to maintain the same posture for a long time. Therefore, 10 mg of morphine can be taken half an hour before SBRT to relieve the patient’s pain and help complete treatment.

Concurrent administration of systemic therapy and SBRT was avoided. SBRT was delivered between chemotherapy cycles, or systemic therapy was held temporarily during SBRT in patients who were undergoing systemic therapy.

In this study, patients can simultaneously receive ≥ 1 planned courses of SBRT to treat ≥ 1 tumors. If the disease had progressed on follow-up surveillance imaging, and met one of the above SBRT indications, the patient will continue to receive a second or subsequent line of SBRT.

### Outcomes and Follow Up

The first year after the SBRT was completed, follow-up was conducted every three months. From the second year to the fifth year, the assessment was conducted every six months, and the follow-up is conducted annually after five years. Treatment results and side effects based on clinical examination, laboratory examination, and CT, MRI, bone scan, or PET-CT were evaluated. The National Cancer Institute’s Common Terminology Standards for Adverse Events (CTCAE) version 4.03 was used to assess toxicity. Acute toxicity is an event that occurs within 90 days of SBRT. Late toxicity is defined as an event that occurs more than 90 days after the start of SBRT.

OS is the time from the start of SBRT to the day of death or the last follow-up, with those lost to follow-up being censored. PFS is defined as any progression or death from the beginning of SBRT, with those lost to follow-up being censored. Local progression (LP) is defined as tumor progression within the irradiated field from the start of SBRT, with those lost to follow-up being censored. The death without LP is a competitive event. The distant relapse is defined as relapse occurring outside the irradiated volume from the start of SBRT. The definition of starting or changing systemic therapy (SCST) is the start of any systemic therapy after SBRT in patients who did not receive systemic therapy or a switch to another systemic therapy after SBRT in patients received systemic therapy. In addition, the death without the event is a competing event. Polymetastatic disease (PMD) was defined as the occurrence of >5 new metastatic lesions from the start of SBRT in oligometastases group. Death without PMD was a competitive event. The visual analog scale (VAS) was used to score pain, and it was divided into four groups according to the score: score 0 for the painless group, score 1–3 for the mild pain group, score 4–6 for the moderate pain group, score 7–10 is the severe pain group. Some patients who progressed after the first-line SBRT received second-line SBRT. PFS2 for these patients is calculated from the start of second-line SBRT.

In patients using SBRT to treat more than one lesion at the same time, PTV is the sum of all individual PTVs to better reflect tumor burden. Assuming an alpha/beta ratio of 10, the average biologically effective dose (BED) is the lowest individual average PTV BED to reflect the lowest dose delivered to a specific tumor.

### Statistical Analyses

We compared numerical variables with normal distribution by *t* test. The nonparametric Wilcoxon test was used when non-normal distribution was found. In order to compare the three groups, ANOVA was used to compare numerical variables with normal distribution and the Kruskal Wallis test when non-normal distribution was found. The Chi-square test with Fisher’s correction for categorical variables was used to compare groups. Kaplan-Meier method and Cox proportional hazard model were used for PFS and OS. Only variables with *p* < 0.05 from the univariate analyses were explored in the multivariate analyses. According to the results of multiple Cox regression analysis, we developed a nomogram (27, 28) by using the rms software package. The discrimination performance was measured by Concordance index (c-index) (29). We assessed the calibration curves by plotting the observed rates against the nomogram-predicted probabilities with 1,000 resamples *via* the bootstrap method.

The accurate of the prognostic prediction were improved as the C-index increased (30). Competitive risk analysis (Gray’s test) (31, 32) can be used to estimate the cumulative incidence of LP for irradiated lesions, cumulative incidences of LP, distant relapse and death occurring as the first event, cumulative incidence of SCST, and cumulative incidence of PMD. All statistical analyses were performed using SPSS 24.0 and R version 3.6.3 (http://www.r-project.org/) for statistical analysis, and *p* < 0.05 was considered statistically significant.

## Results

### Patient Characteristics and SBRT Features

There were a total of 94 patients and 162 lesions treated with SBRT in this paper. 42 patients were in oligometastases (OM) group, 19 patients were in oligoprogression (OP) group, and 33 patients were in local control of dominant tumors (LCDT) group. There were 63 (67.0%) male patients and 31 (33.0%) female patients. The age of the patients in this study ranged from 30 to 85 years (mean age 61 years). The median time between initial diagnosis of colorectal cancer and metastatic disease was 13.2 months (0–90.6 months). After completing the first line of SBRT, the median follow-up time for all patients was 36.4 months. At the last follow-up, 38 patients were lost to follow-up in the study. There were significant differences in the performance status (PS) of Eastern Cooperative Oncology Group among three groups (PS 2–3: OM vs OP vs LCDT, 21.4 vs 47.4 vs 90.9%, *p* = 0.000), as well as the number of metastases (>2 lesions, OM vs OP vs LCDT, 23.8 vs 100 vs 100%, *p* = 0.000) and organs (> 2 organs, OM vs OP vs LCDT s, 0 vs 73.7 vs 97%, *p* = 0.000). The median target size in OM, OP and LCDT groups were 3.0 cm, 4.4 cm and 4.5 cm, respectively (*p* = 0.000). The main characteristics of all patients are summarized in **Table 1**.

**Table 1:** Patient Characteristics.

Variable	Total (n = 94)	Oligometastases (n = 42)	Oligoprogression (n = 19)	Local control of dominant tumors (n = 33)	*P* value
Gender					
Female	31 (33.0%)	12 (28.6%)	6 (31.6%)	13 (39.4%)	0.606
Male	63 (67.0%)	30 (71.4%)	13 (68.4%)	20 (60.6%)	
Age (years), mean (range)	61 (30-85)	64 (48-85)	59 (30-81)	60 (33-77)	0.157
Performance status					
0	10 (10.6%)	9 (21.4%)	1 (5.3%)	0 (0.0%)	0.000
1	36 (38.3%)	24 (57.1%)	9 (47.4%)	3 (9.1%)	
2	37 (39.4%)	9 (21.4%)	8 (42.1%)	20 (60.6%)	
3	11 (11.7%)	0 (0.0%)	1 (5.3%)	10 (30.3%)	
Primary site					
Right Colon	24 (25.5%)	8 (19.0%)	2 (10.5%)	14 (42.4%)	0.065
Left Colon	26 (27.7%)	13 (31.0%)	5 (26.3%)	8 (24.2%)	
Rectum	44 (46.8%)	21 (50.0%)	12 (63.2%)	11 (33.3%)	
Time to metastases^§^(months), median (range)	13.2 (0-90.6)	17.7 (0-90.6)	12.3 (0-41.9)	8.7 (0-58.7)	0.095
Number of lines of previous systemic therapy					0.056
0-1	61 (64.9%)	33 (78.6%)	10 (52.6%)	18 (54.5%)	
2	24 (25.5%)	8 (19.0%)	7 (36.8%)	9 (27.3%)	
3-4	9 (9.6%)	1 (2.4%)	2 (10.5%)	6 (18.2%)	
Pre-SBRT CEA (ug/L), median (range)	20.5 (2-1065)	12.6 (2-1001)	9.0 (2-410)	43 (2-1065)	0.014
Number of metastases					
1	22 (23.4%)	22 (52.4%)	0 (0.0%)	0 (0.0%)	0.000
2	10 (10.6%)	10 (23.8%)	0 (0.0%)	0 (0.0%)	
3	11 (11.7%)	7 (16.7%)	3 (15.8%)	1 (3.0%)	
4	15 (16.0%)	3 (7.1%)	5 (26.3%)	7 (21.2%)	
5	3 (3.2%)	0 (0.0%)	2 (10.5%)	1 (3.0%)	
6	13 (13.8%)	0 (0.0%)	5 (26.3%)	8 (24.2%)	
> 6	20 (21.3%)	0 (0.0%)	4 (21.1%)	16 (48.5%)	
Number of organs involved					
1	29 (30.9%)	29 (69.0%)	0 (0.0%)	0 (0.0%)	0.000
2	19 (20.2%)	13 (31.0%)	5 (26.3%)	1 (3.0%)	
3	27 (28.7%)	0 (0.0%)	12 (63.2%)	15 (45.5%)	
> 3	19 (20.2%)	0 (0.0%)	2 (10.5%)	17 (51.5%)	
Brain metastases					
Yes	13 (13.8%)	0 (0.0%)	0 (0.0%)	13 (39.4%)	0.000
No	81 (86.2%)	42 (100.0%)	19 (100.0%)	20 (60.6%)	
Prior local therapy					
No	71 (75.5%)	34 (81.0%)	13 (68.4%)	24 (72.7%)	0.497
Yes	23 (24.5%)	8 (19.0%)	6 (31.6%)	9 (27.3%)	
Time from metastases to SBRT^※^ (months), median (range)	8.1 (0.2-73.1)	2.8 (0.2-26.1)	12.5 (0.23-73.1)	11.7 (0.23-62.0)	0.000
Metastases in other organs not treated with SBRT					
No	61 (64.9%)	9 (21.4%)	19 (100.0%)	33 (100.0%)	0.000
Yes	33 (35.1%)	33 (78.6%)	0 (0.0%)	0 (0.0%)	
Treated site					
Lung	27 (28.7%)	16 (38.1%)	9 (47.4%)	2 (6.1%)	0.000
Liver	22 (23.4%)	13 (31.0%)	6 (31.6%)	3 (9.1%)	
Brain	12 (12.8%)	0 (0.0%)	0 (0.0%)	12 (36.4%)	
Lymph node	24 (25.5%)	11 (26.2%)	4 (21.1%)	9 (27.3%)	
Other	9 (9.6%)	2 (4.8%)	0 (0.0%)	1 (3.0%)	
Number of metastases treated with SBRT same time					
1	73 (77.7%)	38 (90.4%)	13 (68.4%)	22 (66.7%)	0.069
2	13 (13.8%)	2 (4.8%)	4 (21.1%)	7 (21.2%)	
3-5	8 (8.5%)	2 (4.8%)	2 (10.5%)	4 (12.1%)	
Target size (cm), median (range)	3.6 (1.2-15.1)	3.0 (1.2-5.0)	4.4 (1.3-10.9)	4.5 (2.0-15.1)	0.000
PTV volume (cc), median (range)	30.0 (4-1233.4)	20.8 (4-74)	57.1 (7.6-613.1)	43.3 (12-1233.4)	0.000
PTV coverage (%), median (range)	92.7 (41.1-99.3)	95.2 (66.5-96.9)	86.1 (58.4-95.9)	87.1 (41.1-99.3)	0.001
BED(Gy), median (range)	100 (33.6-180)	109.1 (37.5-180)	105.6 (48-180)	68.4 (33.6-124.8)	0.000

SBRT, stereotactic body radiotherapy; CEA, carcino-embryonic antigen; PTV, planning tumor volume; BED, biological effective dose; Gy, gray.

^§^The time between initial diagnosis of colorectal cancer and of metastatic disease.

^※^The time between initial diagnosis of metastatic disease and SBRT.

Single internal organ was treated with SBRT in 88 (93.6%) patients. Majority of patients (74/94, 78.7%) were treated on one lesion. After the conversion of dose according to BED_10_, 53 lesions (42.4%) were treated with more than 100 Gy. The doses and fractions that varied with metastatic site were summarized in **Supplementary Table 1**. The median prescription isodose was 77%. The duration of treatment was 3–9 days. The median PTV volumes for OM, OP and LCDT groups were 20.8 cm^3^ (in the range of 4.0–74 cm^3^), 57.1 cm^3^ (in the range of 7.6–613.1 cm^3^), and 43.3 cm^3^ (in the range of 12–1,233.4 cm^3^), respectively (*p* = 0.000). The median percentage of PTV coverages in OM, OP and LCDT groups were 95.2% (in the range of 66.5–96.9%), 86.1% (in the range of 58.4–95.9%) and 87.1% (in the range of 41.1–99.3%), respectively(*p* = 0.001). Median BED_10_ was 109.1 Gy of OM, 105.6 Gy of OP and 68.4 Gy of LCDT, respectively (*p* = 0.000). The SBRT features of all patients were summarized in **Table 1**.

### Survival and Prognostic Factors

**Figure 1:**
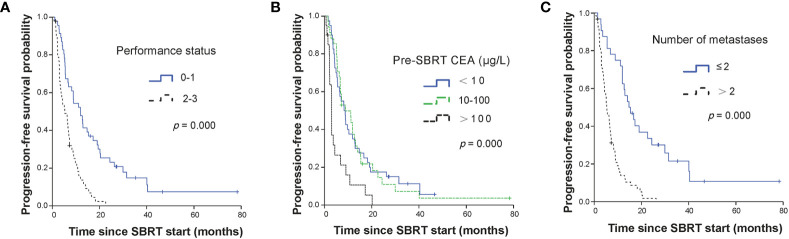
Kaplan-Meier curves for PFS stratified by independent prognostic factors. Panel **(A)** showed performance status; panel **(B)** showed CEA level before SBRT; and panel **(C)** showed the number of metastases. PFS, progression-free survival; SBRT, stereotactic body radiotherapy; CEA, carcino-embryonic antigen.

The median PFS was 7.0 months (95% CI, 4.87–9.13 months) for all patients. The median PFS were 12.6 months (95% CI, 10.12–15.14 months), 6.8 months (95% CI, 5.71–7.89 months) and 3.7 months (95% CI, 2.61–4.86 months) for OM, OP and LCDT groups, respectively. The rates of PFS at 1 year were 52.4% (95% CI, 39.26–69.9), 22.3% (95%CI, 9.4–52.9) and 7.3% (95%CI, 1.99–26.5) for OM, OP and LCDT groups, respectively. For patients who received second-line SBRT at the time of progression (18/94, 19.1%), the median PFS2 was 8.8 months (95% CI, 2.9–14.7 months) for all patients. In univariate analysis, indication (*p* = 0.000), performance status (*p* = 0.000), pre-SBRT CEA (*p* = 0.000), number of metastases (*p* = 0.000), number of organs involved (*p* = 0.000), time from metastases to SBRT (*p* = 0.000), number of metastases treated with SBRT same time (*p* = 0.042), PTV volume (*p* = 0.047), BED (*p* = 0.003) were significant factors for PFS (**Supplementary Table 2**). In multivariable analysis, performance status (0–1 vs 2–3, HR 1.86, 95%CI 1.10–3.12, *p* = 0.020), pre-SBRT CEA (< 10 ug/L vs > 100 ug/L, HR 2.08, 95%CI 1.16–3.73, *p* = 0.013), number of metastases (≤ 2 vs > 2, HR 2.76, 95%CI 1.56–4.89, *p* = 0.001) still were significant factors for PFS (**Table 2**, **Figure 1**).

**Table 2:** Multivariable analysis of PFS and OS.

Co-variates	Category	PFS		OS	
		HR (95% CI)	p-value	HR (95% CI)	p-value
Indication					0.000
	Oligometastases	–	–	Ref.	
	Oligoprogression	–	–	1.07 (0.45-2.56)	0.871
	Local control of dominant tumors	–	–	7.22 (2.99-17.46)	0.000
Performance status	0-1	Ref.		Ref.	
	2-3	1.86 (1.10-3.12)	0.020	3.51 (1.68-7.33)	0.001
Pre-SBRT CEA, micrograms/L			0.025		0.019
	< 10	Ref.		Ref.	
	10-100	0.99 (.603-1.61)	0.952	0.95 (0.49-1.86)	0.885
	> 100	2.08 (1.16-3.73)	0.013	2.60 (1.25-5.39)	0.011
Number of metastases	≤2	Ref.		–	–
	> 2	2.76 (1.56-4.89)	0.001	–	–
PTV volume (cc)	≤30	–	–	Ref.	
	> 30	–	–	3.69 (1.95-7.00)	0.000

PFS, progression-free survival; OS, overall survival; CEA, carcino-embryonic antigen; SBRT, stereotactic body radiotherapy; PTV, planning tumor volume.

**Figure 2:**
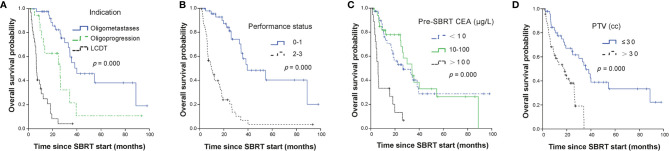
Kaplan-Meier curves for OS stratified by independent prognostic factors. Panel **(A)** showed treatment indication; panel **(B)** showed performance status; panel **(C)** showed CEA level before SBRT and panel **(D)** showed PTV volumes. OS, overall survival; SBRT, stereotactic body radiotherapy; LCDT, local control of dominant tumors; CEA, carcino-embryonic antigen; PTV, planning tumor volume.

The median OS for all patients was 26.1 months (95% CI, 19.35–32.79 months). The median OS of OM, OP and LCDT groups were 40.0months (95% CI, 21.48–58.52), 26.1months (95% CI, 8.08–44.06), and 6.5months (95% CI, 5.50–7.44), respectively. The OS rates for OM group at 1, 2, and 3 years were 97.5% (95%CI, 92.78–100), 82.3% (95%CI, 70.37–96.3), 55.0% (95%CI, 39.02–77.5), respectively. The OS rates for OP group at 1, 2, and 3 years were 75.3% (95%CI, 56.93–99.6), 62.7% (95%CI, 43.04–91.5), 21.5% (95%CI, 6.63–69.8), respectively. The OS rates for LCDT group at 1, 2, and 3 years were 29.1% (95%CI, 16.5–51.3), 8.3% (95%CI, 2.26–30.5) and 4.2% (95%CI, 0.62–27.8), respectively. By univariate analysis, the following factors were significant prognostic variables for OS: indication (*p* = 0.000), performance status (*p* = 0.000), primary site (*p* = 0.033), number of lines of previous systemic therapy (*p* = 0.012), pre-SBRT CEA (*p* = 0.000), number of metastases (*p* = 0.000), number of organs involved (*p* = 0.000), time from metastases to SBRT (*p* = 0.009), treated site (*p* = 0.000), number of metastases treated with SBRT same time (*p* = 0.021), target size (*p* = 0.001), PTV volume (*p* = 0.000), BED (*p* = 0.000) (**Supplementary Table 2**). By multivariable analysis, indication (OM vs LCDT, HR 7.22, 95%CI 2.99–17.46, *p* = 0.000), performance status (0–1 vs 2–3, HR 3.51, 95%CI 1.68–7.33, *p* = 0.001), pre-SBRT CEA (< 10 ug/L vs > 100 ug/L, HR 2.60, 95%CI 1.25–5.39, *p* = 0.011), PTV (≤ 30cc vs > 30cc, HR 3.69, 95%CI 1.95–7.00, *p* = 0.000) were independently significant factors for OS (**Table 2**, **Figure 2**).

### Prognostic Nomogram for OS

**Figure 3:**
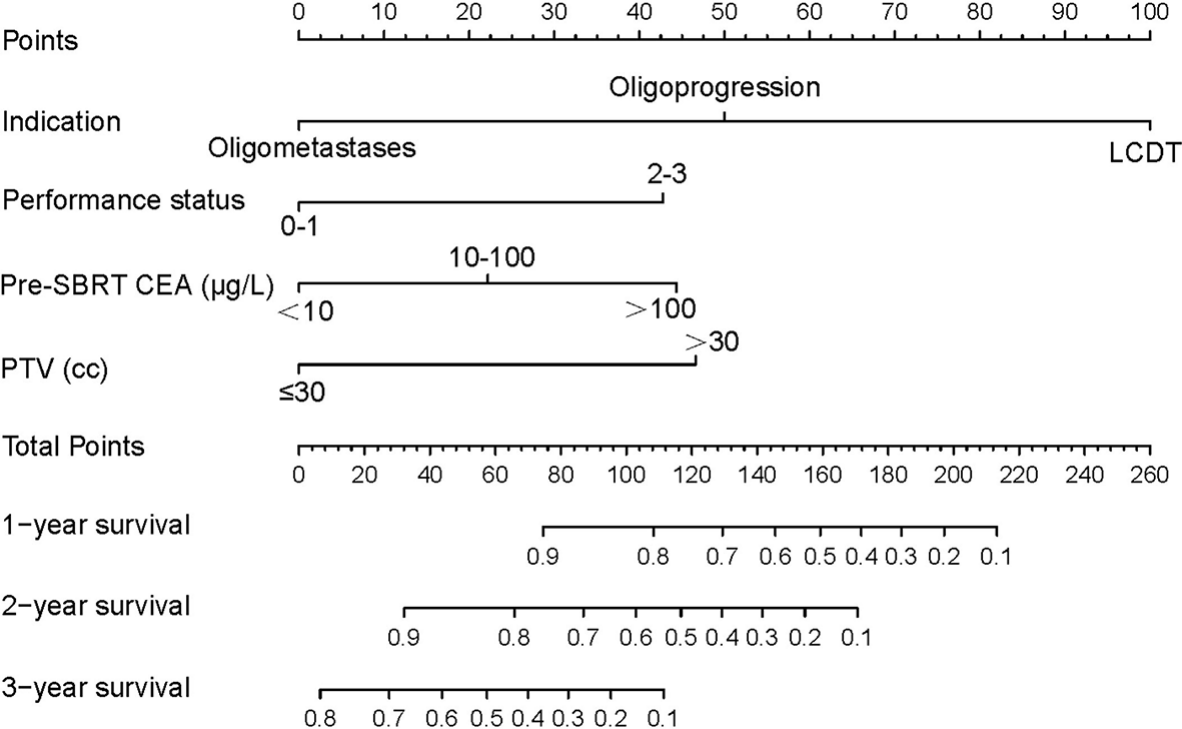
mCRC survival nomogram. (To use the nomogram, the value of each patient was on each variable axis, and a line was drawn upward to determine the number of points received for each variable value. The sum of these numbers was on the Total Points axis. A line was drawn downward to the survival axes to determine the likelihood of 1, 2- or 3-year survival). mCRC, metastatic colorectal cancer; SBRT, stereotactic body radiotherapy; LCDT, local control of dominant tumors; CEA, carcino-embryonic antigen; PTV, planning tumor volume.

**Figure 4:**
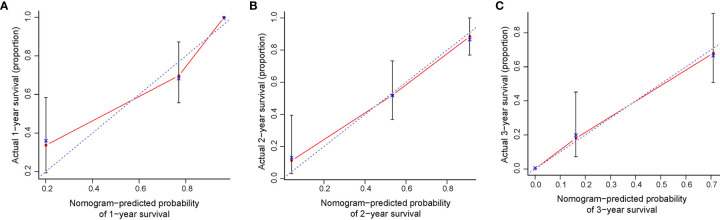
The calibration curve for predicting patient survival at **(A)** 1 year and **(B)** 2 years and **(C)** 3 years. Nomogram-predicted probability of OS was plotted on the x-axis; actual OS was plotted on the y-axis. OS, overall survival.

All significant prognostic factors (Indication, performance status, pre-SBRT CEA and PTV) of OS were identified and integrated to develop a nomogram, as shown in **Figure 3**. The nomogram illustrated indication as sharing the greatest contribution to prognosis, followed by the PTV, pre-SBRT CEA and performance status. Each subtype within these variables was assigned a score on the point scale. A straight line can be drawn to determine the estimated probability of survival at each time point by adding up the total score and locating it on the total point scale. The C-index for OS prediction was 0.848 (95% CI, 0.81 to 0.89). The calibration plot for the probability of survival at 1, 2 or 3 year after SBRT showed a superb agreement between the prediction by nomogram and actual observation (**Figure 4**).

### Cumulative Incidence of LP and Relapse

**Figure 5:**
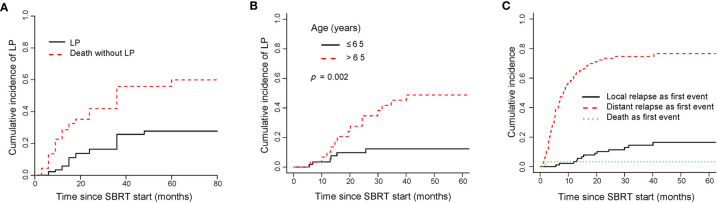
Cumulative incidence curves showed the probability of LP and first events after SBRT. Panel **(A)** showed cumulative incidence of LP; panel **(B)** showed cumulative incidence of LP according to age; panel **(C)** showed cumulative incidence of first events in the entire cohort. LP, local progression; SBRT, stereotactic body radiotherapy.

LP was observed in 20 out of 125 metastases. The median time to LP was 15.5 months in the entire cohort. The cumulative incidences of LP for all patients were 5.9% (95%CI, 2.15–12.3), 16.4% (95%CI, 9.19–25.47) and 25.7% (95%CI, 16.21–36.3) at 1, 2, and 3 years, respectively (**Figure 5A**). The cumulative incidences of LP for the OM, OP and LCDT groups were 21.8% (95%CI,10.05–36.4), 12.6% (95%CI, 1.81–34.19) and 11.4% (95%CI,1.1–20.9) at 2-year, respectively. Univariate analysis revealed that age (*p* = 0.002), indication (*p* = 0.029), number of lines of previous systemic therapy (*p* = 0.04) and number of organs involved (*p* = 0.015) were correlated to LP (**Supplementary Table 3**). By multivariate analysis, older patients (≤65 years vs > 65 years, subdistribution HR=3.68, 95%CI 1.35–10.03, *p* = 0.011) were associated with higher rates of LP (**Figure 5B**).

For the entire cohort, relapse was more likely to occur outside the irradiated field than within it (**Figure 5C**). The rate of distant relapse as a first event was higher than that of local relapse as a first event. By univariate analysis, indication (*p* = 0.000), performance status (*p* = 0.002), number of lines of previous systemic therapy (*p* = 0.014), number of metastases (*p* = 0.000), number of organs involved (*p* = 0.000), time from metastases to SBRT (*p* = 0.016), target size (*p* = 0.03) were associated with the rate of distant relapse as a first event (**Supplementary Table 3**). No significant prognostic factor for the rate was found by multivariate analysis.

### Cumulative Incidence of SCST

**Figure 6:**
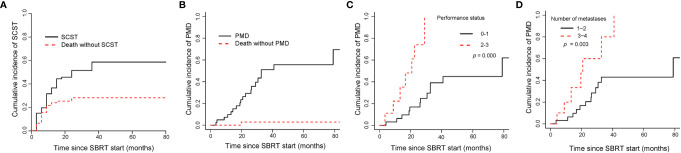
Cumulative incidence curves showed the probability of SCST and PMD. Panel **(A)** showed cumulative incidence of SCST, and **(B)** showed cumulative incidence of PMD in the entire cohort; panel **(C, D)** showed cumulative incidence of PMD in oligometastases group according to performance status and number of metastases. SCST, starting or changing systemic therapy; PMD, polymetastatic disease; SBRT, stereotactic body radiotherapy.

The median time to SCST for all patients was 7.4 months. In the whole group, the cumulative incidences of SCST were 36.61% (95%CI, 26.48–46.77), 51.51% (95%CI, 39.98–61.88) and 58.65% (95%CI, 46.34–69.07) at 1, 2, and 3 years, respectively. The cumulative incidences of SCST for the OM, OP and LCDT groups were 28.3% (95%CI, 15.07–43.1), 50.5% (95%CI, 22.77–72.92) and 40.9% (95%CI, 22.62–58.42) at 1 year, respectively. No statistically significant variables affected the cumulative incidence of SCST in univariate analysis. The cumulative incidence curve of SCST showed the probability of each competition event in the entire cohort (**Figure 6A**).

### Cumulative Incidence of PMD in OM Patients

The median time to PMD was 20.8 months in OM group. The cumulative incidences of PMD were 9.7% (95%CI, 3.03–21.08), 29.22% (95%CI, 15.52–44.37) and 51.06% (95%CI, 31.78–67.42) at 1, 2, and 3 years, respectively (**Figure 6B**). Gender (*p* = 0.042), PS (*p* = 0.000) and number of metastases (*p* = 0.003) were correlated to PMD in univariate analysis. In multivariate analysis, PS (0–1 vs 2–3, subdistribution HR=5.49, 95%CI 2.2–13.7, *p* = 0.000, **Figure 6C**) and number of metastases (1–2 vs 3–4, subdistribution HR=2.45, 95%CI 1.05–5.72, p = 0.038, **Figure 6D**) were significant factors in the cumulative incidence of PMD. PMD after SBRT was significantly associated with shorter OS (2-year OS rate, 94.1% versus 69.3%; *p* = 0.000).

### Pain Relief of LCDT Patients

Prior to SBRT, 28 (84.9%) patients in the local control of the dominant tumors group had pain in different parts of the body. There were 14, 9, and 5 cases of mild pain, moderate pain, and severe pain, respectively. After SBRT, 100% of patients experienced various degrees of pain relief, including 26 patients with no or mild pain, and 2 patients with moderate pain, within 2 weeks after SBRT. Before SBRT, the median VAS score was 3.5, and after SBRT, the median VAS score droped significantly to 1.0 (*p* = 0.000).

### Toxicity

Overall, treatments were well tolerated with no case of therapy-related death. In terms of acute toxicity, the most frequent side effects were fatigue (16/94, grade 1/2 reactions), nausea (14/94, grade 1), anorexia (11/94, all grade 1), which normalized within 3 months after SBRT. For overall hematological toxicity, cases of grades 1, 2 and 3 account for 12.8% (12/94), 6.4% (6/94) and 1.1% (1/94) of total cases, respectively. Transient chest pain and intestinal obstruction that required medication were each observed in 1 patient. In addition, one patient treated with lung SBRT (45 Gy/2 fractions). After two months, he presented with grade 2 toxicity caused by acute radiation pneumonitis. The symptoms were resolved following conservative measures. There were no grade 4 or 5 toxicities. No late toxicity was observed.

## Discussion

This study showed that SBRT offered favorable survival, disease control and symptom palliation for mCRC patients, and the proposed nomogram could provide individual prediction of OS for these patients. Some studies have compared the efficacies of SBRT among OM, OP, and LCDT (21, 33). The survival results of our study were consistent with other reports that mCRC patients with OM had the highest median OS and PFS after SBRT compared with OP or LCDT. The comparison of three groups subject to an inherent selection bias, because LCDT patients have many poorer prognostic features, poorer performance status, a greater number of metastases and more involved organs. Some large retrospective case series of mCRC for SBRT have suggested prognostic variables for survival. Favorable prognostic factors included good performance status (18, 34), fewer metastases (18, 34), smaller tumors (5, 21, 34, 35), fewer number of lines of previous systemic therapy (5, 21), lower CEA (21), and oligometastasis treatment indication (18, 21). Our multivariate analysis demonstrates that treatment indication, performance status, pre-SBRT CEA along with PTV were significant independent variables for survival.

Previous studies have reported the role of nomogram to predict survival specifically for mCRC (36, 37). Renfro et al. (38) constructed a validated clinical nomogram to quantify the risk of early death after initial treatment of mCRC. And the C-index for 90-day mortality prediction was 0.77. Sjoquist et al. (39) built prognostic nomograms for 1-year OS and 6-month PFS in mCRC by using the ARCAD database. However, the prognostic nomogram for long-term survival outcomes after SBRT in mCRC were scarce. Hence, we combined the known clinical variables to establish a nomogram for individual patients during SBRT of mCRC. The developed nomogram herein evaluated survival using indication, performance status, pre-SBRT CEA and PTV. Our nomogram was quantitative with good prognostic efficiency. It is convenient for clinicians and patients to quantitate OS in the pretreatment setting. Risk estimates by the model can guide clinical decision making and patient counseling, especially in the discussion of less aggressive treatment options or additional supportive care with patients at a more advanced stage of their mCRC disease timeline. The internal validation of our model showed agreement between the calibration plots and satisfactory c-indices.

It is reasonable to evaluate the local control of CRC metastases. In this paper, the cumulative incidence of LP was only 5.9% at 1 year after SBRT for the entire cohort. In the present analysis, there was no correlation between dose according to BED_10_ and local control. However, most of lesions (42.4_10_) were treated with a BED_10_ value higher than 100 Gy. Regarding patterns of recurrence, the first relapse in the entire cohort was more likely to occur outside the irradiated field than within it. Those with OP/LCDT status were more likely to relapse at distant sites, perhaps because these patients have greater systemic involvement from the outset. Therefore, SBRT in progressive treatment has different goals. The focus was not on survival, but on relieving symptoms and delaying systemic treatment.

Another interesting endpoint in this study is the cumulative incidence of SCST which affects both physicians and patients. In some cases, the next line of treatment may have significantly toxic, or the options for systemic therapy may be limited. Compared with other more invasive options, SBRT may be cost effective with minimal adverse effect on life quality of patients. Some retrospective studies reported that SBRT was used to delay the change of systemic therapy in colorectal cancer (21), non-small cell lung cancer (33, 40) and pulmonary metastases (41). A randomized phase II study showed that the local ablative therapy (surgery or SBRT) significantly delayed the start of androgen deprivation therapy for patients with oligorecurrent prostate cancer compared with surveillance alone for oligorecurrent prostate cancer (42). In our study, after 1 year of SBRT for the entire cohort, the cumulative incidence of SCST was 36.61%. During this period, many patients do not need to change the systemic therapy strategy. Ultimately, we can delay the demand to change systemic therapy by using SBRT. In addition, our study showed that the median PFS2 of 8.8 months after the second SBRT was in line with the median PFS1 of 7.2 months after the first SBRT. This suggests that subsequent “lines” of repeated SBRT have cumulative benefits for patients to delay further progression.

We also explored the role of SBRT in postponing the conversion to PMD (43, 44), which is not ameanable anymore of local treatment. Nicosia et al. (45) reported that the median time to PMD was 25.8 months in CRC patients with lung oligometastases after receiving SBRT. They confirmed that SBRT can postpone the transition to PMD. In the present study, the median time to PMD was 20.8 months in the OM group. After SBRT, the 2-year OS rates were 94.1 and 69.3% for patients remained OM and patients with PMD, respectively. Thus, it is important to keep patients in an oligometastatic state for as long as possible.

For mCRC, reducing symptoms such as pain was considered to be the major goal of improving life quality of patients. Wang et al. (46) reported significant pain reduction during the first 6 months after SBRT for managing spinal metastases. In another study, 80 mCRC patients with symptomatic pelvic mass were treated with palliative radiotherapy, and the pain palliation was observed in 79% of the cases (47). Our study showed that 100% of patients achieved pain relief after SBRT. For the 28 patients who experienced pain before radiotherapy, the pain VAS score was reduced after treatment. SBRT has a significant pain-relieving effect, which can reduce or resolve pain or decrease analgesia, thereby improving the life quality of patients.

This study is a single-arm retrospective study from a single-center. The study is mainly limited by small sample size, broad lesion size and radiation schedules. In addition, the data is heterogeneous in metastatic sites, radiation sites and treatment before SBRT. It is difficult to review all late toxicities, such as radiation pneumonitis or bone fracture after SBRT for lung or for bone, due to retrospective study. In the future, high quality prospective trials are needed to determine the specific benefit that SBRT offers in different subsets of patients, tumors and clinical settings.

## Conclusions

In conclusion, mCRC patients who are not suitable for metastasectomy have good survival after SBRT, with reduced symptoms and relatively low risk of toxicity. In addition, a novel nomogram is established and validated for predicting survival of patients with mCRC for SBRT, which may help to tailor individualized treatment.

## Data Availability Statement

The raw data supporting the conclusions of this article will be made available by the authors, without undue reservation.

## Ethics Statement

The studies involving human participants were reviewed and approved by the Ethics Committee of Jinling Hospital, and written informed consents were obtained from all patients. The patients/participants provided their written informed consent to participate in this study.

## Author Contributions

XC and CC designed the study. XJ and ZS collected the data. XJ and XZ wrote the manuscript. AL and XZ analyzed and interpreted the data. All authors contributed to the article and approved the submitted version.

## Funding

This work was supported by grants from National Natural Science Foundation of China (81872042 and 81972333), Foundation of Jiangsu Province (BK20181238), and Jiangsu Postdoctoral Research Foundation (1701156C).

## Conflict of Interest

The authors declare that the research was conducted in the absence of any commercial or financial relationships that could be construed as a potential conflict of interest.

## Abbreviations

BED, biologically effective dose; C-index, concordance index; CEA, carcino-embryonic antigen; CT, computed tomography; CI, confidence interval; CTCAE, Common Terminology Standards for Adverse Events; ECOG, Eastern Cooperative Oncology Group; ^18^F-FDG-PET/CT, 18-fluorodeoxyglucose positron emission tomography/computed tomography; GTV, gross tumor volume; Gy, Gray; HR, hazard ratio; LP, local progression; mCRC, metastatic colorectal cancer; MRI, magnetic resonance imaging; OS, overall survival; PFS, progression-free survival; PMD, polymetastatic disease; OM, oligometastases; OP, oligoprogression; LCDT, local control of dominant tumors; PTV, planning tumor volume; PS, performance status; RTOG, Radiation Therapy Oncology Group; SBRT, stereotactic body radiotherapy; SCST, starting or changing systemic therapy; SABR, stereotactic ablative radiotherapy; VAS, visual analog scale.
