# Supplementary material for: Outcomes of Stereotactic Body Radiotherapy for Metastatic Colorectal Cancer With Oligometastases, Oligoprogression, or Local Control of Dominant Tumors
Source: Front Oncol. 2021 Jan 29;10:595781. doi: 10.3389/fonc.2020.595781 (PMC7878536; doi:10.3389/fonc.2020.595781)
Supplement: Supplementary file 1 [file Table_1.docx]

| **Supplementary Table 1. Dose fractionation schedules in different metastatic site.** | | | | |
| --- | --- | --- | --- | --- |
| Variable | Prescribed dose (Gy) | No. of fractions | BED_10_ | No. of lesions |
| Lung | 40 | 4 | 80 | 1 |
|  | 48 | 6 | 86.4 | 2 |
|  | 48 | 4 | 105.6 | 2 |
|  | 50-60 | 5 | 100-132 | 9 |
|  | 45-60 | 3 | 112.5-180 | 15 |
|  | 40-45 | 2 | 120-146.25 | 5 |
| Liver | 35 | 5 | 59.5 | 3 |
|  | 49 | 7 | 83.3 | 1 |
|  | 48 | 6 | 86.4 | 1 |
|  | 50 | 5 | 100 | 1 |
|  | 48 | 4 | 105.6 | 9 |
|  | 45-51 | 3 | 112.5-137.7 | 14 |
| Spine/bone | 21-39 | 3 | 35.7-89.7 | 6 |
|  | 32 | 4 | 57.6 | 1 |
|  | 45 | 5 | 85.5 | 1 |
| Brain | 30-36 | 3 | 60-79.2 | 11 |
|  | 32-36 | 4 | 57.6-68.4 | 4 |
|  | 36-42 | 6 | 57.6-71.4 | 2 |
|  | 28 | 2 | 67.2 | 1 |
| Lymph node | 24 | 6 | 33.6 | 1 |
|  | 25-45 | 5 | 37.5-85.5 | 21 |
|  | 40-44 | 4 | 80-92.4 | 3 |
|  | 36 | 3 | 79.2 | 1 |
|  | 50 | 5 | 100 | 1 |
|  | 48 | 4 | 105.6 | 3 |
|  | 42-48 | 3 | 100.8-124.8 | 3 |
| Adrenal | 50 | 5 | 100 | 1 |
| Abdominal wall | 33-36 | 3 | 69.3-79.2 | 2 |
| Median | 45 | 4 | 100 | - |
| ***Abbreviations:*** BED, biological effective dose; Gy, gray. | | | | |
