# Supplementary material for: Outcomes of Stereotactic Body Radiotherapy for Metastatic Colorectal Cancer With Oligometastases, Oligoprogression, or Local Control of Dominant Tumors
Source: Front Oncol. 2021 Jan 29;10:595781. doi: 10.3389/fonc.2020.595781 (PMC7878536; doi:10.3389/fonc.2020.595781)
Supplement: Supplementary file 2 [file Table_2.docx]

| **Supplementary Table 2. Univariate analysis of PFS and OS.** | | | | | |
| --- | --- | --- | --- | --- | --- |
| Co-variates | Category | PFS | | OS | |
|  |  | Median PFS (95% CI) in months | p-value | Median OS (95% CI) in months | p-value |
| Indication | Oligometastases | 12.6 (10.12-15.14) | 0.000 | 40.0 (21.48-58.52) | 0.000 |
|  | Oligoprogression | 6.8 (5.71-7.89) |  | 26.1 (8.08-44.06) |  |
|  | Local control of dominant tumors | 3.7 (2.61-4.86) |  | 6.5 (5.50-7.44) |  |
| Gender | Female | 6.5 (3.66-9.34) | 0.574 | 24.9 (15.33-34.47) | 0.388 |
|  | Male | 7.2 (5.05-9.35) |  | 26.1 (16.28-35.86) |  |
| Age (years) | ≤ 65 | 6.7 (5.11-8.23) | 0.325 | 24.9 (15.71-34.09) | 0.524 |
|  | > 65 | 9.0 (4.93-13.13) |  | 26.6 (16.28-36.98) |  |
| Performance status | 0-1 | 11.9 (7.44-16.30) | 0.000 | 39.3 (18.55-60.11) | 0.000 |
|  | 2-3 | 5.3 (2.61-7.99) |  | 9.7 (5.15-14.25) |  |
| Primary site | Right Colon | 5.3 (2.54-8.06) | 0.087 | 7.0 (0.00-20.08) | 0.033 |
|  | Left Colon | 6.3 (4.50-8.04) |  | 19.8 (1.97-37.63) |  |
|  | Rectum | 10.3 (6.66-14.00) |  | 34.0 (24.01-44.05) |  |
| Time to metastases | ≤ 24 | 6.8 (5.23-8.37) | 0.153 | 26.1 (18.10-34.04) | 0.503 |
|  | > 24 | 10.3 (4.22-16.44) |  | 29.9 (10.50-49.36) |  |
| Number of lines of previous systemic therapy | ≤ 1 | 8.2 (5.25-11.09) | 0.057 | 33.0 (23.08-42.86) | 0.012 |
|  | > 1 | 6.6 (4.82-8.38) |  | 16.6 (6.41-26.85) |  |
| Pre-SBRT CEA(µg/L) | < 10 | 8.3 (5.33-11.21) | 0.000 | 26.6 (6.20-47.06) | 0.000 |
|  | 10-100 | 8.8 (3.92-13.74) |  | 34.0 (23.78-44.28) |  |
|  | > 100 | 3.0 (2.90-3.10) |  | 6.0 (3.79-8.21) |  |
| Number of metastases | ≤ 2 | 14.7 (9.76-19.64) | 0.000 | 88.9 (28.77-149.09) | 0.000 |
|  | > 2 | 5.3 (3.73-6.87) |  | 13.4 (6.19-20.67) |  |
| Number of organs involved | ≤ 2 | 11.9 (9.75-13.99) | 0.000 | 38.3 (30.62-45.92) | 0.000 |
|  | > 2 | 4.8 (3.38-6.28) |  | 10.4 (7.10-13.76) |  |
| Prior local therapy | No | 8.2 (5.92-10.42) | 0.201 | 26.1 (17.14-35.12) | 0.305 |
|  | Yes | 6.3 (4.81-7.73) |  | 18.2 (3.46-32.94) |  |
| Time from metastases to SBRT (months) | ≤ 12 | 9.0 (5.68-12.38) | 0.000 | 29.9 (19.75-40.11) | 0.009 |
|  | > 12 | 5.4 (4.32-6.42) |  | 18.2 (5.65-30.75) |  |
| Treated site | Lung | 8.2 (4.78-11.56) | 0.185 | 34.0 (24.34-43.72) | 0.000 |
|  | Liver | 9.0 (0.00-18.39) |  | 34.2 (14.27-54.19) |  |
|  | Lymph node | 7.2 (4.52-9.88) |  | 24.9 (13.35-36.45) |  |
|  | Other | 4.3 (3.34-5.26) |  | 9.7 (4.10-15.30) |  |
| Number of metastases treated with SBRT same time | 1 | 8.8 (6.24-11.42) | 0.042 | 26.6 (15.98-37.28) | 0.021 |
|  | 2-5 | 6.8 (4.67-8.93) |  | 9.7 (4.57-14.83) |  |
| Target size (cm) | ≤ 3 | 10.3 (6.29-14.37) | 0.090 | 38.3 (30.83-45.71) | 0.001 |
|  | > 3 | 6.6 (4.77-8.43) |  | 18.2 (14.71-21.69) |  |
| PTV volume (cc) | ≤ 30 | 8.3 (5.62-10.92) | 0.047 | 35.6 (27.91-43.29) | 0.000 |
|  | > 30 | 6.5 (4.59-8.41) |  | 18.2 (9.64-26.82) |  |
| PTV coverage | ≤ 90% | 5.6 (3.58-7.56) | 0.135 | 22.2 (5.92-38.54) | 0.408 |
|  | > 90% | 8.2 (5.82-10.52) |  | 26.8 (12.25-41.41) |  |
| BED (Gy) | ＜100 | 5.3 (3.08-7.52) | 0.003 | 13.4 (5.43-21.43) | 0.000 |
|  | ≥ 100 | 9.0 (4.14-13.80) |  | 34.2 (27.68-40.78) |  |
| ***Abbreviations:*** PFS, progression-free survival; OS, overall survival; CEA, carcino-embryonic antigen; SBRT, stereotactic body radiotherapy; PTV, planning tumor volume; BED, biological effective dose; Gy, gray. | | | | | |
