# Supplementary material for: Outcomes of Stereotactic Body Radiotherapy for Metastatic Colorectal Cancer With Oligometastases, Oligoprogression, or Local Control of Dominant Tumors
Source: Front Oncol. 2021 Jan 29;10:595781. doi: 10.3389/fonc.2020.595781 (PMC7878536; doi:10.3389/fonc.2020.595781)
Supplement: Supplementary file 3 [file Table_3.docx]

| **Supplementary Table 3. Univariate analysis of LP and first event distant relapse.** | | | |
| --- | --- | --- | --- |
| **Co-variates** | **Category** | **Local progression** | **First event distant relapse** |
|  |  | p-value | p-value |
| Indication | Oligometastases | 0.029 | 0.000 |
|  | Oligoprogression |  |  |
|  | Local control of dominant tumors |  |  |
| Gender | Female | 0.96 | 0.25 |
|  | Male |  |  |
| Age | ≤ 65 | 0.002 | 0.051 |
|  | > 65 |  |  |
| Performance status | 0-1 | 0.31 | 0.002 |
|  | 2-3 |  |  |
| Primary site | Right Colon | 0.68 | 0.017 |
|  | Left Colon |  |  |
|  | Rectum |  |  |
| Time to metastases | ≤ 24 | 0.57 | 0.45 |
|  | > 24 |  |  |
| Number of lines of previous systemic therapy | ≤1 | 0.04 | 0.014 |
|  | > 1 |  |  |
| Pre-SBRT CEA(µg/L) | < 10 | 0.31 | 0.26 |
|  | 10-100 |  |  |
|  | > 100 |  |  |
| Number of metastases | ≤2 | 0.21 | 0.000 |
|  | > 2 |  |  |
| Number of organs involved | ≤2 | 0.015 | 0.000 |
|  | > 2 |  |  |
| Prior local therapy | No | 0.49 | 0.08 |
|  | Yes |  |  |
| Time from metastases to SBRT (months) | ≤12 | 0.2 | 0.016 |
|  | > 12 |  |  |
| Treated site | Lung | 0.55 | 0.075 |
|  | Liver |  |  |
|  | Lymph node |  |  |
|  | Other |  |  |
| Number of metastases treated with SBRT same time | 1 | 0.92 | 0.45 |
|  | 2-5 |  |  |
| Target size (cm) | ≤ 3 | 0.13 | 0.03 |
|  | > 3 |  |  |
| PTV volume (cc) | ≤ 30 | 0.56 | 0.96 |
|  | > 30 |  |  |
| PTV coverage | ≤ 90% | 0.91 | 0.49 |
|  | > 90% |  |  |
| BED (Gy) | ＜100 | 0.52 | 0.063 |
|  | ≥ 100 |  |  |
| ***Abbreviations:*** PFS, progression-free survival; OS, overall survival; CEA, carcino-embryonic antigen; SBRT, stereotactic body radiotherapy; PTV, planning tumor volume; BED, biological effective dose; Gy, gray. | | | |
